# p53/NF-kB Balance in SARS-CoV-2 Infection: From OMICs, Genomics and Pharmacogenomics Insights to Tailored Therapeutic Perspectives (COVIDomics)

**DOI:** 10.3389/fphar.2022.871583

**Published:** 2022-05-27

**Authors:** Daniela Milani, Lorenzo Caruso, Enrico Zauli, Adi Mohammed Al Owaifeer, Paola Secchiero, Giorgio Zauli, Donato Gemmati, Veronica Tisato

**Affiliations:** ^1^ Department of Translational Medicine, University of Ferrara, Ferrara, Italy; ^2^ Department of Environmental and Prevention Sciences, University of Ferrara, Ferrara, Italy; ^3^ Department of Research, King Khaled Eye Specialistic Hospital, Riyadh, Saudi Arabia; ^4^ Ophthalmology Unit, Department of Surgery, College of Medicine, King Faisal University, Al-Ahsa, Saudi Arabia; ^5^ Centre Haemostasis and Thrombosis, University of Ferrara, Ferrara, Italy

**Keywords:** SARS-CoV-2, p53, NF-kB, ACE2, cytokine-storm, omics, pharmacogenomics, ACE1

## Abstract

SARS-CoV-2 infection affects different organs and tissues, including the upper and lower airways, the lung, the gut, the olfactory system and the eye, which may represent one of the gates to the central nervous system. Key transcriptional factors, such as p53 and NF-kB and their reciprocal balance, are altered upon SARS-CoV-2 infection, as well as other key molecules such as the virus host cell entry mediator ACE2, member of the RAS-pathway. These changes are thought to play a central role in the impaired immune response, as well as in the massive cytokine release, the so-called cytokine storm that represents a hallmark of the most severe form of SARS-CoV-2 infection. Host genetics susceptibility is an additional key side to consider in a complex disease as COVID-19 characterized by such a wide range of clinical phenotypes. In this review, we underline some molecular mechanisms by which SARS-CoV-2 modulates p53 and NF-kB expression and activity in order to maximize viral replication into the host cells. We also face the RAS-pathway unbalance triggered by virus-ACE2 interaction to discuss potential pharmacological and pharmacogenomics approaches aimed at restoring p53/NF-kB and ACE1/ACE2 balance to counteract the most severe forms of SARS-CoV-2 infection.

## Introduction

Severe acute respiratory syndrome coronavirus-2 (SARS-CoV-2) is the highly transmissible virus responsible for the ongoing coronavirus disease 2019 (COVID-19) pandemic. The host-pathogen interaction occurs within the viral spike protein and the human membrane receptor angiotensin-converting enzyme 2 (ACE2) with the involvement of the transmembrane serine protease 2 (TMPRSS2) (Hoffmann et al., 2020; Zhao et al., 2020; Kaplan et al., 2021). As main functional receptor for SARS-CoV-2, ACE2 has been detected in variety of human tissues (Bartolak-Suki et al., 2021; Li and Qin, 2021) including enterocytes, ciliated cells both in upper and lower airways, type II alveolar pneumocytes **(** Zhao et al., 2020; Bartolak-Suki et al., 2021) and conjunctival and corneal cells (Kitazawa et al., 2021; Ma et al., 2021; Sasamoto et al., 2021 **)**. ACE2-virus interaction results into unrestrained ACE2 downregulation (Hoffmann et al., 2020) leading to alteration of key physiological pathways and overall resulting in the clinical spectrum of COVID-19.

The balance between ACE2 activity and that of its counterpart ACE1 represents the core of the renin-angiotensin-system (RAS) in which ACE2 mediates the conversion of angiotensin-II (Ang-II) into Ang1-7, a vasodilator factor involved in the controlling of local tissue homeostasis by anti-inflammatory, anti-coagulant, anti-proliferative and anti-fibrotic activities (Bourgonje et al., 2020; Gemmati and Tisato, 2020). Alteration in ACE2 activity due to viral infection leads to a cascade of biological and pathological outcomes characterizing COVID-19 pathophysiology with the compromise of the respiratory tract and of other organs including cardiovascular damage, renal and gastrointestinal injury as well as increased incidence of thromboembolic events (Bourgonje et al., 2020). The pathophysiological picture includes a sustained series of complications experienced by a proportion of COVID-19 patients and commonly referred as “long COVID”, characterized but not limited to fatigue, shortness of breath, and cognitive dysfunction affecting the daily quality of life and basic everyday activities (Soriano et al., 2021). In this light long COVID, which is often used as synonymous of “post-acute sequelae of SARS-CoV-2 infection” (PASC), is dramatically emerging as secondary epidemic event that need to be properly addressed in terms of pathophysiological insights and potential treatment strategies. As reported in a recent systematic review (2100 studies in which 57 met inclusion criteria) accounting for 250.351 COVID-19 survivors, 54.0% of survivors experienced at least one PASC after 1 month, and 55.0%, 54.0% of patients experienced at least one PASC after 2–5 and 6 months after infection respectively (Groff et al., 2021). With regard to non-hospitalized COVID-19 patients, a recent report on a cohort of 445 participants SARS-CoV-2 PCR-positive test highlighted persistent symptoms on 36% of symptomatic subjects at 4 weeks follow-up, mainly associated with female sex and body mass index **(** Bliddal et al., 2021). Although the pathophysiology of PASC is still unknown, several evidence-supported mechanistic hypotheses can be considered including long-lasting hyperinflammation triggered by the persistence of viral particles in tissues and organs; abnormal innate immune response and immune stimulation; interfering effects with autoantibodies; mitochondrial dysfunction and metabolic changes **(** Nalbandian et al., 2021; Merad et al., 2022; Su et al., 2022 **)**. Recent data seems to support beneficial effect of anti-SARS-CoV-2 vaccinations on long COVID, with a lower susceptibility and a lowering effect on syndrome severity and life impact **(** Antonelli et al., 2022; Tran et al., 2022). Potential mechanisms underlying these positive effects might be related to a vaccine-induced clearance of viral antigens or to a more balanced and regulated inflammatory response, though dedicated clinical studies are needed to identify the pathways involved.

Since the start of the ongoing pandemic, there has been a massive effort to identify risk factors for disease susceptibility and worst prognosis. In this light, host genetics, either in terms of ethnicity and individual genetic predispositions, emerged as strongly involved in the different clinical heterogeneity of COVID-19 (Severe Covid-19 GWAS Group, 2020; COVID-19 Host Genetics Initiative, 2021; Nakanishi et al., 2021). A recent GWAS found significant COVID-19 association signals at locus 3p21.31 (rs11385942), comprising the solute carrier *SLC6A20* (Na+ and Cl-coupled transporter family), and at locus 9q34.2 (rs657152) coincident with the *ABO* blood group (rs8176747, rs41302905, rs8176719) in severe COVID-19 patients and interestingly, both loci are pharmacogenetically associated to the RAS-pathway **(** Chung et al., 2010; Severe Covid-19 GWAS Group, 2020). Of note, the lead variant (rs11385942) is located in an intergenic region spanning several genes (*SLC6A20, LZTFL1, CCR9, FYC O 1, CXCR6, XCR1*) and at least two of them (*LZTFL1, CCR9*) are closely located with the risk GA-allele of rs11385942 associated with increased expression in human lung cells of *SLC6A20* and *LZTFL1*. Subsequent studies confirmed the relevance of the 3p21.31 locus in COVID-19 susceptibility and prognosis considering the presence of ancient DNA introgressed from Neanderthal populations **(** Zeberg and Paabo, 2020, 2021; Huffman et al., 2022) and highlighted additional genetic loci as well as rare variants involved in disease predisposition and prognosis **(** Benetti et al., 2020; COVID-19 Host Genetics Initiative, 2021; Nakanishi et al., 2021; Schmiedel et al., 2021; Fallerini et al., 2022 **)**. Moreover, in a sex and gender perspective, male sex together with old age and presence of critical comorbidities including diabetes, hypertension and cardiovascular disease have been considered among the strongest features accounting for large part of the severe cases (Bonaccorsi et al., 2020; Gemmati et al., 2020). In detail, people older than 60 suffer the worst outcome with higher mortality rates greater than 50% with increased fatality rates in males compared to females as also demonstrated for earlier SARS **(** Chen and Subbarao, 2007; Casimir et al., 2013; Channappanavar et al., 2017).

In terms of pathophysiological mechanisms insights, it is of interest that several recent reports highlight the potential interfering effects of exogenous infections (i.e. SARS-Cov-2) on human endogenous retroviruses (HERVs), that may undergo activation leading in turn to inflammatory and immune responses (Durnaoglu et al., 2021). During COVID-19 infection, genes belonging to different HERVs families (i.e. HERV-H, HERV-K, HERV-W) have been found upregulated in CD4 and CD8 lymphocytes of patients in a disease severity-related manner, and correlated to inflammatory markers such as IL-6, IL-17, TNF-α, CCL-2, CXCL6 (Balestrieri et al., 2021; Garcia-Montojo and Nath, 2021). Moreover, in the context of COVID-19 pediatric patients, Tovo and others reported a differential expression of IFN-I and -II, TRIM28, SETDB1 and HERVs in the presence of severe and mild clinical phenotypes, suggesting a role of HERVs in influencing the course of the disease **(** Tovo et al., 2021).

As far as therapeutic approaches, several treatments were tested in the attempt of controlling symptoms and complications including antiviral drugs or alternative approaches (Tiwari Pandey et al., 2020; Gil Martinez et al., 2021 **)**, convalescent plasma administration (Salazar et al., 2021), therapeutic antibodies (Baum et al., 2020; Liu et al., 2020) and the newly-derived antiviral pills under clinical evaluation (Ledford, 2021). Nonetheless, the pandemic evolution has significantly changed when different vaccine formulations became available, and it is expected that vaccines will be indeed the decisive key for virus eradication. However, despite a massive vaccination program has been put in place in the last year, there are several concerns that need to be considered. Firstly, the appearance of new “variants of concern” (i.e. virus variants showing a significant impact on transmissibility, severity and/or immunity) (ECDC, 2022) may in part invalidate the vaccine-induced protection. Again, there are several difficulties in putting in place a comprehensive global vaccination because of logistic and economic issues as well as individual attitudes standing opposition to vaccinations. In this scenario, the efforts in optimizing vaccination strategies need to be associated with efforts in establishing “ready-to-use” therapeutic strategies for symptomatic patients for which a definite cure is still missing. In this regard, much effort has been recently made to elucidate the molecular mechanisms and intracellular pathways mostly involved in both SARS-CoV-2 infection and COVID-19 pathophysiological processes; also by using bioinformatics approaches **(** Alnajeebi et al., 2022; Hasankhani et al., 2021; Kwan et al., 2021; Rahaman et al., 2021). These studies have shown that genes related to immune and apoptosis-related functions were deregulated, and further analysis revealed that p53 signaling was one of the most enriched pathway upon SARS-CoV-2 infection. Moreover, additional studies emphasized the role of single nucleotide polymorphism (SNP) of p53 on host immune response against the virus as a potential predictor of the outcome in SARS-CoV-2 infected patients (Lodhi et al., 2021).

p53 is commonly referred as the guardian of genome, mainly acting on cell-cycle control through the cyclin G1/Mdm2/p53 axis (Gordon et al., 2018). In the context of this review, it is remarkable that immune and DNA repair systems are interdependent (Bednarski and Sleckman, 2019). For example, loss of function of key DNA repair proteins lead to defects in the production of functional B and T mature cells **(** Difilippantonio et al., 2008; Bednarski and Sleckman, 2019; Jiang and Mei, 2021). On the other hand, virus-mediated DNA damage occurs through ROS and oxidative stress effects on host cells (Delgado-Roche and Mesta, 2020; Cardozo and Hainaut, 2021; Jiang and Mei, 2021) and the persistence of DNA damage represents a harmful additional contributing factor to viral infection-induced pathology. In this respect, by using *in vitro* cell models it has been recently demonstrated that SARS-CoV-2 is able to inhibit DNA damage repair by affecting the recruitment of crucial cell cycle regulators and DNA repair as BRCA1 and p53-binding protein-1 (53BP1), overall affecting adaptive immunity (Indraccolo et al., 2006; Jiang and Mei, 2021 **)**.

As the immune response modulation in different organs is concerned (Agupitan et al., 2020; Levine, 2020; Chang et al., 2021; Shi and Jiang, 2021), p53 is now regarded as guardian of the whole immune integrity, crucial in the context of SARS-CoV-2 **(** Major et al., 2020; Saini et al., 2020). The wide range of p53 biological activities requires a stringent regulation of the protein itself, since it represents a central hub in the signal transduction of various cellular processes involved in tissue homeostasis and metabolic response controls (Lahalle et al., 2021), as well as in cancer development and progression (Nagpal and Yuan, 2021). In the context of viral infections, it is therefore not surprising that p53 deficiency makes the host more susceptible and permissive to infections (Ma-Lauer et al., 2016). Among p53 regulatory networks, firstly we have to consider that the half-life of p53 is very short (about 15–20 min). Then, the physiological p53 basal levels are kept low in most normal tissues by its principal regulator, the Mouse Double Minute 2 (MDM2) human homolog, also known as E3 ubiquitin-protein ligase (Tisato et al., 2017). Recent evidence indicates that also the basal levels of p53 are physiologically relevant, since p53 basal levels suppression is associated with disruption of tissue homeostasis (Cui et al., 2021). Thus, in spite of the suggestion that p53 basal levels are in normal conditions maintained low, basal p53 functions are now considered a key regulatory issue on tissue homeostasis by controlling anabolic cell metabolism (Nagpal and Yuan, 2021). Of note, low p53 levels have been associated with severe respiratory disorders, suggesting a protective role of p53 in vascular homeostasis and lung inflammation **(** Uddin and Barabutis, 2020).

**FIGURE 1:**
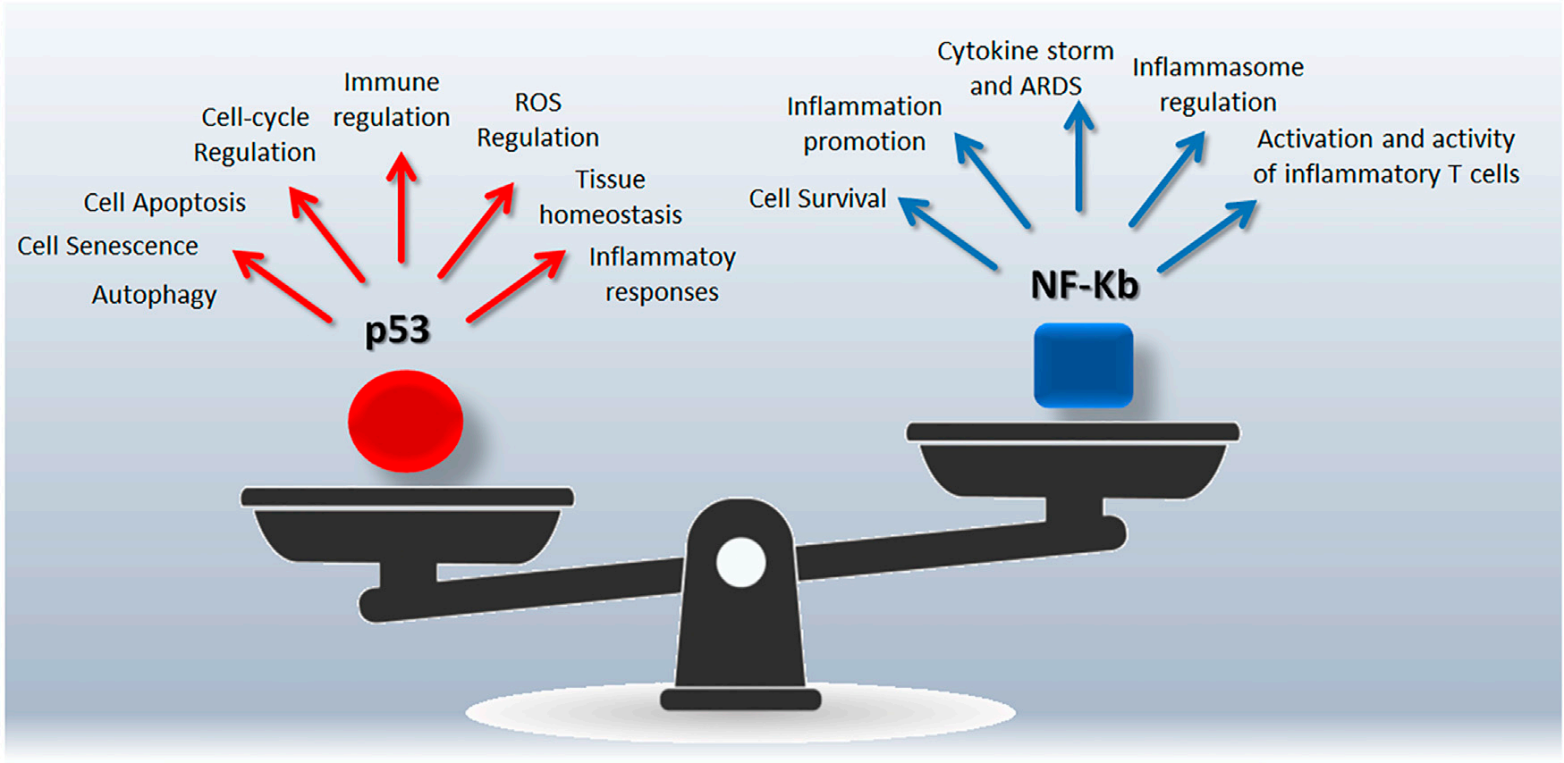
Snapshot of the p53/NF-Kb unbalancing during SARS-CoV-2 infection. Both p53 and NF-Kb are hub responsible for complex signalings leading to different biological outcomes.

NF-κB (nuclear factor κB) is a collective acronym referred to a transcription factors family involved in the regulation of genes belonging to immune and inflammatory pathways (including cytokines, chemokines, inflammasome) (Liu et al., 2017), as well as to cell proliferation and survival (Van Antwerp et al., 1996). NF-κB activation occurs *via* two major pathways, referred as the canonical and alternative pathways, in response to several stimuli as pathogens, inflammation, ligands of the TNFR superfamily (Liu et al., 2017). The canonical activation of NF-κB leads to phosphorylation, ubiquitination, and proteolytic degradation of IκB (NF-κB family inhibitors) allowing the translocation of NF-κB to the nucleus (Karin and Ben-Neriah, 2000). The alternative pathway is activated by specific stimuli and depends on the processing of p100 (NF-κB2 precursor protein, also known as p52) (Liu et al., 2017). In the frame of the host-pathogen relationship, there is a tight connection between virus infection and NF-κB pathway that emerges as specific target of several human viruses (Santoro et al., 2003). In this line, upregulation of most NF-κB signaling pathway genes has been reported in COVID-19 patients and associated to hyperinflammatory phenotype (Hirano and Murakami, 2020; Sohn et al., 2020). The complexity of the NF-κB pathway arises from intricate mechanisms regulating its functions, including feedback loops and crosstalk with other pathways such as p53 and RAS signaling able to regulate cell responses to extracellular stimuli as well as inflammation and immune response (Perkins, 2007). This integrated view supports the concept of a synergic targeting of p53 and NF-κB to manage COVID-19 and maximize the effectiveness of treatments (Figure 1).

### SARS-CoV-2 and p53 Interaction

Starting from the assumption that a number of different viruses have established strategies to escape the p53-mediated control of the cell cycle and of antiviral immunity, as far as SARS-CoV-2 is concerned we can identify two levels of interactions that can be potentially targeted by dedicated pharmacological interventions.

A direct interplay between p53 and SARS-CoV-2 has been suggested for the first time following an *in silico* study reporting on the ability of the S2 subunit of the virus to physically and strongly interact with p53 **(** Singh and Bharara Singh, 2020). In the same line, another mechanism of interaction has been highlighted in a large animal model study showing that p53 can regulate ACE2 receptor in a tissue- and sex-specific fashion (Zhang et al., 2021). It is of interest that the reported higher expression of ACE2 in females can be related to estrogens activity, and perhaps contributing to the lower disease susceptibility of females compared to males (Bonaccorsi et al., 2020). Thus, this first level of interactions between p53 and SARS-CoV-2 occurs *via* the modulation of ACE2 expression in a sex-related manner and *via* a direct interaction between the S2 subunit of the viral spike protein and p53, although the significance of this interaction has not been addressed so far. Of note, a direct regulatory activity of p53 on SARS-CoV-2 replication is a shared feature characterizing other previous coronaviruses (Ma-Lauer et al., 2016). In detail, by using a high-throughput yeast-2-hybrid methodology, Ma-Lauer and others focused on the molecular mechanisms of coronaviruses–host interactions, demonstrating that pathogens counteract the antiviral activity of p53 through the stabilization of the ring finger and CHY zinc finger domain containing 1 (RCHY1) who mediates p53 degradation (Ma-Lauer et al., 2016). The viral SARS-unique domain (SUD) and papain-like protease (PLpro) emerged as the key viral-mediators of the interaction.

A second and more relevant level of interaction between SARS-CoV-2 and p53 is represented by the ability of SARS-CoV-2 to up-regulate the major p53 inhibitor MDM2 (Klein et al., 2021). During SARS-CoV replication process, there is the synthesis of two polyproteins (pp1a and pp1ab) that after additional cleavage by the papain-like protease (PLpro) and 3C-like protease (3CLpro) are converted into 16 nonstructural proteins (nsps), of which 15 constituting the viral replication and transcription complex (RTC) (V'Kovski et al., 2021). PLpro exhibits several functions other than the viral proteins processing. These additional functions mainly converge towards viral evasion of host immune system *via* deubiquitinating and deISG15ylating (interferon-induced gene 15) activity (Yan and Wu, 2021), highlighting a multifunctional nature of PLpro involved in both viral cycle and viral survival (Osipiuk et al., 2021). With regard to PLpro deubiquitinating activity, it has been shown that when the deubiquitinated substrate is the host MDM2, the overall result is a stabilization of MDM2 itself leading in turn to accelerated proteasome degradation of basal p53 (Dong et al., 2020). These data suggest that part of the survival strategy of SARS-CoV-2 is based on the down-regulation of the basal levels of p53, which in turns leads to perturbation of the tissue homeostasis, as above-mentioned (Cui et al., 2021).

In this frame, it is of interest the analysis performed by Yan and Wu exploring an apparent contradiction on the comprehensive role of PLpro by using insight on SARS-CoV that could be nonetheless translated to SARS-CoV-2. As the authors suggested, p53 ubiquitination is useful from a virus-perspective allowing viral replication, on the other hand, E3 stabilization leads to a biological cascade (e.g. ubiquitination of IkBa, TRAFs, STING) culminating with the activation of innate antiviral immune responses (Yan and Wu, 2021). The strategy proposed is a differential sequence of events mediated by PLpro according to kinetic preference, potentially applicable to SARS-CoV-2 with antiviral therapeutic potential (Yan and Wu, 2021).

### Role of NF-kB in Promoting SARS-CoV-2 Infection

It has been clearly shown that SARS-CoV-2 triggers an inflammatory response characterized by activation of NF-kB, which represents a key element in the well described “cytokine storm” observed in patients affected by SARS-CoV-2 (Sallenave and Guillot, 2020 **)**. In particular, the essential role of NF-kB in mediating SARS-CoV-2 disease features has been shown in a series of recent studies **(** Hemmat et al., 2021; Bartolini et al., 2022), outlining the role of endoplasmic reticulum stress and NF-kB in SARS-CoV-2 infected cells. An interesting approach was followed by Do and others (Do et al., 2020), who aimed to address the epidemiological evidence that COVID-19 is more severe in adults than in children. In the attempt of finding biological reasons underlying this phenomenon, the authors analyzed potential differences in ACE2 expression and differences in the immune responses to viral infection. Of note, they proposed that NF-kB might be the hub of different biological outcomes involved in the severe clinical manifestations of COVID-19 in adults, from hyperinflammation up to coagulation/thrombotic processes affecting in particular platelets and endothelial cells (Mussbacher et al., 2019; Do et al., 2020). In this context, complications in children affected by “pediatric inflammatory multisystem syndrome temporally associated with SARS-CoV-2” might also be associated to NF-kB activity. Overall, the comparison of virological and immunological data from COVID-19 patients at different age suggests the presence of an “over-reactive” immune response in adults compared to children. It is of particular interest the analysis performed by the authors to identify a potential link between clinical features of COVID-19 in children and those of children affected by Kawasaki Disease, a rare condition characterized by autoimmune systemic vasculitis, sharing a common hyper-inflammatory state and an over activation of NF-kB p65 during disease acute phase (Do et al., 2020).

In terms of mechanistic insights, it is of interest that SARS-CoV nucleocapsid protein (N) mediated IL-6 expression occurs *via* the direct binding of N protein on NF-κB promoter and *via* facilitation of the nuclear translocation of NF-κB **(** Zhang et al., 2007). NF-kB is an important regulator of proinflammatory cytokine production and B-cell function, and it has been reported that there is a clear link between altered basal NF-κB and the pro-inflammatory hallmarks of aging and age-associated diseases (Bektas et al., 2014). The central role of the NF-κB pathway in COVID-19 may therefore partially explain the severest disease phenotype experienced by elderly and by patients affected by comorbidities such as obesity and diabetes characterized by chronic NF-κB upregulation (Kircheis et al., 2021). In the presence of such “altered condition”, the virus-host interaction further increases NF-κB levels *via* different mechanisms, including Angiotensin II (AngII) accumulation following virus-mediated ACE2 downregulation that leads to additional NF-κB activation (Brasier et al., 2000; Pandey et al., 2015).

Recent evidence has shown that SARS-CoV-2 infection, already following the interaction of its spike proteins with the surface of target cells, induces a potent inflammatory response **(** Attiq et al., 2021; Khan et al., 2021; Kircheis et al., 2021). It has been recently demonstrated by epigenetic and single-cell transcriptomic analyses that a selective NF-κB signature was most prominent in SARS-CoV-2 infected cells (Nilsson-Payant et al., 2021). NF-κB signaling disturbance by silencing of NF-κB transcription factor p65 or p50 caused loss of virus replication, suggesting that the vigorous cytokine release after SARS-CoV-2 infection is the result of a NF-κB-dependent viral replication. Of note, also conjunctival cells showed a robust induction of NF-kB as well as diminished type I interferon signaling in response to SARS-CoV-2 infection (Eriksen et al., 2021). In a different study (Hariharan et al., 2021) it was confirmed that SARS-CoV-2 activated the NF-kB pathway in various cells, such as macrophages of lung, liver, kidney, central nervous system, gastrointestinal tract and cardiovascular system, and inhibition of NF-kB pathway has been proposed as potential therapeutic action in alleviating the severe forms of SARS-CoV-2.

### Modulation of NF-kB and p53 activity as a strategy against SARS-CoV2

NF-kB and p53 crosstalk can be considered an integrated cell-signalling with therapeutic potential. From one side different stimuli may induce the two pathways, and while p53 induces apoptosis, NF-κB induces resistance to cell death suggesting the presence of a regulatory mechanism to harmonize the cell fate also at the transcriptional level (Perkins, 2007).

The cell “decision-making” is the result of multiple interactions, including the competition for complexes containing the p300/CBP coactivator protein (Webster and Perkins, 1999), and IKK (kinase complex and core element of the NF-κB cascade) interaction, also involved in IκB-α phosphorylation and degradation (Israel, 2010; Carra et al., 2016). Moreover, NF-κB may downregulate p53 *via* the induced expression of MDM2 **(** Tergaonkar et al., 2002; Tergaonkar and Perkins, 2007), or *via* an autoregulatory loop in which NF-κB activation induces BCL3 gene (Kashatus et al., 2006). On the other hand, there is also a cooperative cross talk between the two pathways with a direct regulation of p53 gene by NF-κB (Perkins, 2007). Of note, these reciprocal regulatory interactions represent potential targets to restore p53 normal functions and/or regulate NF-kB activity.

p53 deficiency on severe respiratory disorders has been recently reviewed **(** Uddin and Barabutis, 2020), highlighting opposite roles of p53 and NF-kB in the lungs. Of note, preclinical models clearly indicate that p53 protects from damage-induced lung impairment antagonizing NF-kB mediated inflammatory/immune responses (Uddin and Barabutis, 2020). Moreover, the experience coming from tumor biology suggests that NF-κB activation counterbalance the pro-apoptotic activity of p53 by affecting p53 viability through MDM2 induction (Luo et al., 2005), in line with the similarities between tumorigenesis process and those related to viral infection and host-virus interaction (Mehta et al., 2021).

Several studies have shown that a variety of natural anti-inflammatory compounds **(** Lin et al., 2021; Qin et al., 2021), including polyphenols, well known for their cardioprotective and anti-cancer activity (Chairez-Ramirez et al., 2021), have protective *in vitro* effects also against SARS-CoV-2 infection (De Angelis et al., 2021) and all these compounds decrease the cellular levels of NF-kB. Interestingly, polyphenols have also been shown to up-regulate *TP53* gene expression and protein levels and modulate posttranslational modifications such as phosphorylation, acetylation, and ubiquitination, showing the ability to influence protein stability, subcellular compartment location and activity (Chairez-Ramirez et al., 2021). In this context, the interplay between p53 and NF-kB has been clearly established in a variety of studies **(** Machado et al., 2018; Jang et al., 2020; D'Orazi et al., 2021), and it has also been shown that inhibition of MDM2 abrogates inflammation through NF-κB suppression (Fan et al., 2018).

In line with the different strategies for drug repurposing to target virus replication and disease/complications (Sultana et al., 2020; Smith et al., 2022), we have previously hypothesized that p53 activator Nutlin-3, a potent and selective activator of p53 might be useful to counteract SARS-CoV-2 infection (Zauli et al., 2020). Besides natural compounds with anti-inflammatory activity, other studies have directly suggested that pharmacological activation of p53 might be a strategy to counteract SARS-CoV-2 infection (Ramaiah, 2020; Zauli et al., 2020). A potential beneficial anti-viral mechanism of p53 activation is to reduce the senescence-associated secretory phenotype and to decrease the levels of inflammatory cytokines (Wiley et al., 2018; Huang et al., 2020), at least in part through the inhibition of MDM2. More recently, by using Connectivity Map (CMap) bioinformatics resources to identify drugs that may be repositioned for of COVID-19 treatment, Bonnet and others reported the potential use of anti-cancer agents including small molecules acting as MDM2 regulators as well as anti-inflammatory molecules, signaling and metabolite inhibitors and neuromodulators (Bonnet et al., 2022 **)**.

Interestingly, natural compounds and phytoconstituents have also been considered for their anti-viral functions and potential therapeutic effects in the management of COVID-19 **(** Nallusamy et al., 2021; Ali et al., 2022). Among the others, it has been reported that phillyrin could significantly inhibit SARS-CoV-2 replication *in vitro* **(** Ma et al., 2020a), and it has been shown the ability of reducing the protein expression of NF-kB p65 *in vitro* as well as the release of pro-inflammatory cytokines. Moreover, Liu Shen capsule, a traditional Chinese medicine with detoxification and antiphlogistic activity, possesses antiviral and anti-inflammatory effects against SARS-CoV-2 infection mediated by the suppression of NF-kB signaling pathway (Ma et al., 2020b). In the frame of combined therapies based on the synergic effect of Western and traditional Chinese remedies, it has been recently reported that He-Jie-Shen-Shi decoction (HJSS) may represent an adjuvant therapy, and dedicated network pharmacology studies further identified p53 as one of the target of HJSS combination therapy (Hu et al., 2021).

Finally, in the light of a more “holistic” approach, traditional herbal remedies with multifunctional therapeutic methods may be considered additional strategies to boost the immune system in the general population and strengthen the defenses against infections especially for those subjects under self-confinement or at risk because of old age, by adequate diet and nutrition **(** Iddir et al., 2020; Pandey et al., 2020). There is a clear connection between nutritional quality and immune status, particularly among the elderly often experiencing a decline of immune defenses related to aging together with immune weakening also due to micronutrient deficiencies. Frequently, poor nutrition status is associated with long-lasting subclinical inflammatory and oxidative stress conditions. In this line, there is a growing interest on the preventive and therapeutic potential of dietary constituents with high anti-inflammatory and antioxidant capacity such as vitamin C, vitamin E and phytochemicals, which can suppress NF-kB activity. Dietary fiber, fermented by the gut microbiota into short-chain fatty acids (Iddir et al., 2020; Jabczyk et al., 2021), as well as vitamin D (Xu et al., 2020) have also shown to produce efficient anti-inflammatory effects.

### Genomics insights and Pharmacogenomics interactions: p53/NF-kB and RAS-pathway

In the first phase of pandemic, many drugs utilized to contrast COVID-19 did not yield fully established clinical effectiveness and safety and the repurposing strategy, if pharmacogenetically tuned, could have given better results and additive advantages to any treatment thanks to a more personalized medicine approach. Basically, adverse drug reactions (ADRs) and drug-drug interactions (DDIs) are the most common risks also exacerbated by the polypharmacy condition often present in the elder comorbid patients affected by COVID-19.

A first line approach should consider the Cytochrome P450 (*CYP*) subfamily genes, since many repositioned antivirals or supporting treatments in COVID-19 are metabolized by CYP 450 enzymes. Genetic variants in the *CYP* genes modulating their expression contribute to the individual drug response variability by altering pharmacokinetics and pharmacodynamics of the specific drug. These genes are highly polymorphic (e.g. *CYP3A4/5*, *CYP2B6*, *CYP2C8*, *CYP2C9*, *CYP2C19*, *CYP2D6*) and are characterized by a wide geographic variability (Zhou et al., 2017; Biswas et al., 2020; Sukprasong et al., 2021 **)** interacting with large part of those drugs specifically used in COVID-19 (Sukasem et al., 2013; Biswas et al., 2022). Interestingly, many clinical complications of COVID-19 belong to the same group of comorbidities responsible for the poor prognosis, and drugs acting as anti-inflammatory, antithrombotic (anti-coagulant and anti-aggregant), anti-hypertensive or with lipid-lowering action, have marked pharmacogenomics features resembling those belonging to the lipoproteins and homocysteine homeostasis genes **(** Parmeggiani et al., 2008; Gemmati et al., 2009; Gemmati et al., 2016; Tisato et al., 2019; Cappadona et al., 2021; Tisato et al., 2021; Biswas et al., 2022).

A paradigmatic example, in which pharmacogenomics would have been extremely helpful in sorting out this intricate concern, comes from the debated appropriateness of the use or maintenance of RAS-inhibitors during symptomatic or asymptomatic SARS-CoV2 infection (Mancia et al., 2020; Mehra et al., 2020; Reynolds et al., 2020). Symptomatic COVID-19 patients often have history of hypertension, diabetes, cardiovascular disease, thrombosis, and RAS-inhibitors are among the elective treatment of such patients regardless the presence or not of SARS-CoV2 infection. This point rapidly progressed towards interest on the role of RAS-pathway genes and raised pharmacogenomics concerns on the safety of such pharmacological approach **(** Gemmati et al., 2020; Gemmati and Tisato, 2020). The theoretical worry, i.e. continuing or dismissing RAS-inhibitors (ACEi or ARBs) in COVID-19 patients, was based on the hypothesis that SARS-CoV-2 enters human cells through ACE2 membrane receptor, and such specific treatment could favor in turn viral infection by enhancing ACE2 receptor availability. A wide metanalysis reported that ACEi/ARBs did not worsen COVID-19 severity in treated patients, suggesting on the contrary possible protective effects of RAS-inhibitors in COVID-19 management thanks to a potential pharmacogenomics rebalancing of ACE1/ACE2 equilibrium directly affected by SARS-CoV-2 entry (Gemmati et al., 2020; Gemmati and Tisato, 2020; Guo et al., 2020 **)**.

**FIGURE 2:**
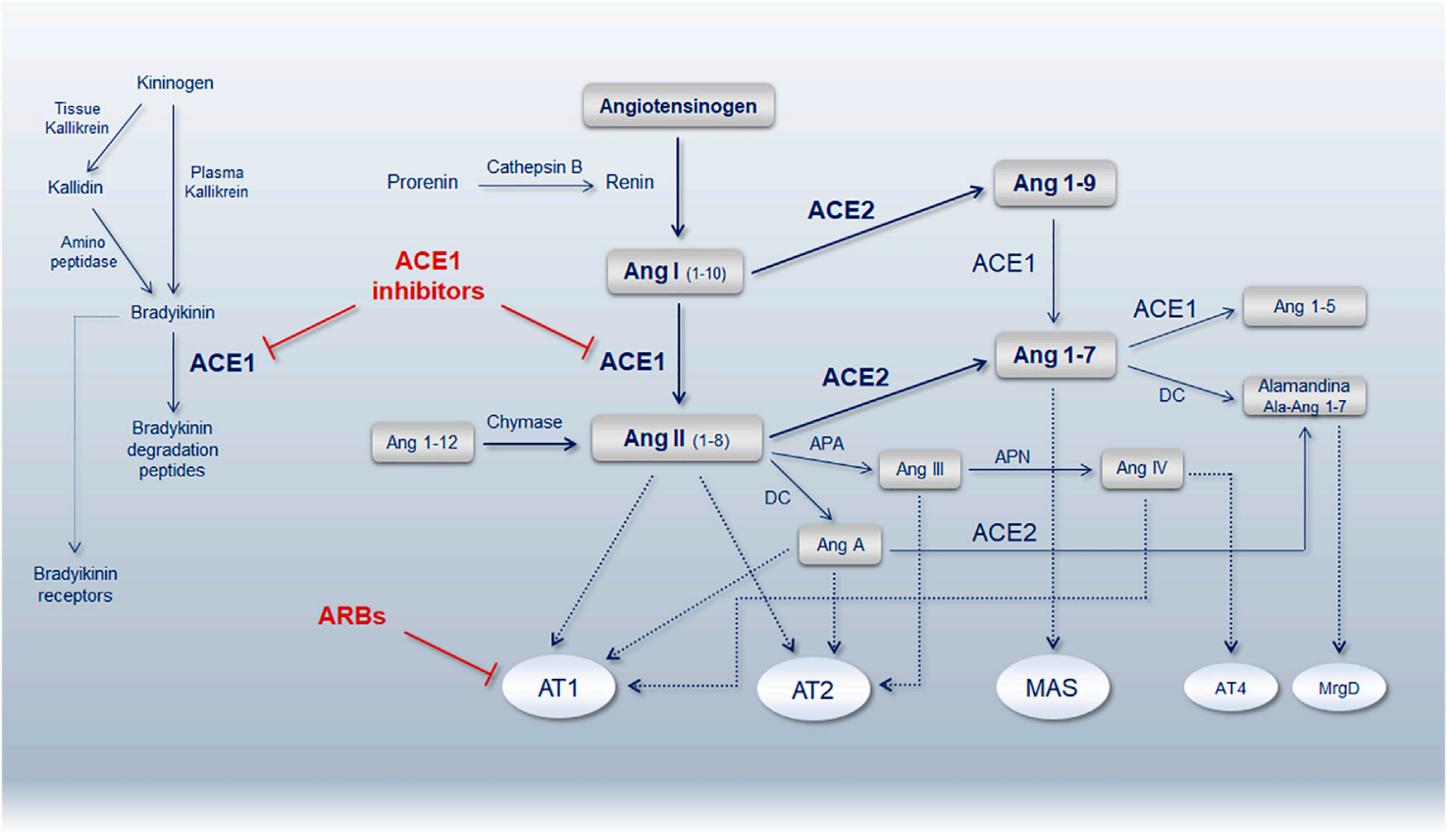
Schematic representation of the renin-angiotensin-system (RAS) pathway showing the main bioactive peptides and key receptors. ACE1, angiotensin-converting enzyme 1; ACE2, angiotensin-converting enzyme 2; Ang, angiotensin; AT1R, Ang-II type 1 receptor; AT2R, Ang-II type 2 receptor; ARBs, angiotensin receptor blockers; MAS, Mas receptor; AT4R, angiotensin receptor type 4; MrgD, MAS-related G protein-coupled receptor member D; APA, aminopeptidase A; APN, aminopeptidase N; DC, decarboxylase (Reproduced from Gemmati et. al. Genes. 11 (9), 1044. doi:10.3390/genes11091044) (Gemmati and Tisato, 2020).

ACE1 (EC 3.4.15.1) and ACE2 (EC 3.4.17.23) are antagonist enzymes involved in opposite pathways, the ACE1/Ang-II/AT1-receptor is a local vasoconstrictor/proliferative and procoagulant axis, while the ACE2/Ang1-7/MAS-receptor is a vasodilator/anti-proliferative and anticoagulant axis (Figure 2). The main activity of these enzymes is significantly driven by common intragenic polymorphisms also characterized by a peculiar geographic distribution that disclosed exciting correspondences between haplotypes distribution and worldwide COVID-19 morbidity and mortality index (Yamamoto et al., 2020).

*ACE1* gene maps on locus 17q23.3 (Gene ID: 1636), its product (ACE1) converts Ang-I to Ang-II. A common insertion/deletion (I/D) of 287-bp in the Alu-sequence of intron 16, includes four individual SNPs (rs4646994, rs1799752, rs4340 and rs13447447) and modulates ACE1 expression (Zhong et al., 2012). Alternative splicing caused by the presence of Alu-sequences, is responsible for the loss of one of the two active enzyme sites in the *ACE1* I-allele, leaving the D-allele counterpart containing the two active sites favoring in turn the Ang-I to Ang-II conversion **(** Purwaningroom et al., 2015; Widodo et al., 2017). Consequently, D/D genotype has the highest ACE1 activity, I/D genotype intermediate levels, and the I/I genotype the lowest ACE1 level (Itoyama et al., 2004).

*ACE2* gene maps on locus Xp22.22 (Gene ID: 59272), its product (ACE2) converts Ang-II to Ang 1–7. Several gene variants in the *ACE2* gene (Smyth et al., 2019), among those amino acids considered crucial for ACE2 stability and SARS-CoV-2 entry, hypothesized sex-differences in receptor-virus affinities (Gemmati et al., 2019; Gemmati et al., 2020). The most relevant change is the transition G8790A (rs2285666), with the G/- (male hemizygote) or G/G (female homozygote) genotypes having about 50% reduced expression compared to A/- or A/A genotypes in male and female respectively **(** Wu et al., 2017; Pinheiro et al., 2019). G8790A substitution is at the beginning of *ACE2* intron 3 (c.439+4G > A) causing alternative splicing mechanisms affecting in turn gene expression (Li, 2012; Yang et al., 2015). Moreover, two additional intronic SNPs (rs1978124 and rs714205) are in strong linkage disequilibrium with rs2285666 influencing haplotyping and genome-phenome associations **(** Benjafield et al., 2004; Huang et al., 2006).

*TP53* gene maps on locus 17p13.1 (Gene ID: 7157), alternative splicing of *TP53* and the presence of alternate promoters result in multiple transcripts and isoforms with different activities. Of note, an intragenic variant in the exon 4 of *TP53* gene results in a Pro-to-Arg substitution (P72R; rs1042522), it was described in the past that the amino acid residue 72 can be changed in Arg, Pro, or Cys, and these changes resulted in distinct isoforms of human p53 molecule (Matlashewski et al., 1987). Recent studies ascribed to the P72-allele a significantly enhanced response to inflammatory stimuli compared to R72-allele, and studies in knock-in humanized p53 mice showed altered degree of NF-kB-dependent apoptosis in P72 or R72 mice **(** Azzam et al., 2011). In addition, P72 and R72 alleles show a dramatically different response to the innate immunity stress also against SARS-CoV-2 infection (Leu et al., 2013; Lodhi et al., 2021). Interestingly, these differences are regulated by NF-kB target genes that are better activated by the P72 variant accounting for intriguing personalized feedbacks in the different populations (Frank et al., 2011).

The hypothesized pharmacogenomics mechanism linking RAS-pathway and p53/NF-kB is based on their mutual equilibrium involved in maintaining tissue and cardio-vasculature integrity. When disturbed, e.g. a genetically unbalanced Ang-I to Ang-II conversion, the major effector of the RAS system (i.e. Ang-II) can cause increased local oxidative stress, vasculature damage and senescence via genomic mechanisms mediated by Ang-II/AT1/Aldosterone interactions causing detrimental effects on several transcription factors including NF-kB and p53 (Min et al., 2009; Husi et al., 2013). In details, *ACE2* downregulation (e.g. due to SARS-CoV-2 - human cell entry) exacerbated by the presence of the loss of function *ACE2* 8790G-allele, and the concomitance of *ACE1* upregulation (e.g. due to the presence of the gain of function *ACE1* D-allele), may lead to unrestrained RAS deregulation. Additive or synergistic or antagonistic effects might come from the coexistence of the P72/R72 alleles in the *TP53* gene characterized by significant differences in response to inflammatory stimuli and to the innate immunity stress against SARS-CoV-2 infection (Azzam et al., 2011; Leu et al., 2013; Lodhi et al., 2021). This hypothesized scenario, causing increased genetic susceptibility (e.g. unrestrained RAS-pathway activation), is not a remote situation due to the high frequency of such variants particularly among those populations characterized by high SARS-CoV-2 case-fatality rate **(** Pinheiro et al., 2019; Asselta et al., 2020; Gemmati and Tisato, 2020).

Inherited predispositions and acquired alterations disturbing RAS-pathway and NFkB-p53 balance may affect anti-inflammatory, anticoagulant, anti-proliferative, anti-fibrotic, anti-apoptotic and anti-oxidant activities, leading to organ dysfunction and failure. ACEi and ARBs show high efficacy in contrasting Ang-II levels to counteract RAS over-activation. Similarly, Idasanutlin by restoring p53 pathway might in turn rebalance p53/NF-kB, contrast virus spreading and favor organ, tissue and vasculature homeostasis recovery. In addition, considering RAS pathway over-activation as one of the main causes in determining COVID-19 severity, is in line with the recently GWAS identified associations of *ABO* and *SLC6A20* loci **(** Severe Covid-19 GWAS Group, 2020), being crucial modifiers of local Ang-II levels and water/salt reabsorption **(** Chung et al., 2010; Terao et al., 2013; Gasso et al., 2014; Ling et al., 2014; Vuille-dit-Bille et al., 2015; Gemmati et al., 2020).

The mutual connections between RAS-genes (i.e. *ACE1*/*ACE2*) and *TP53/NFKB1* genes, and the observation that, as described for *ACE1* and *ACE2* gene variants, the frequencies of R72/P72-allele of *TP53* gene strongly vary among different ethnicities correlating with SARS-CoV2 mortality/infection rates, open promising pharmacogenomics approaches for COVID-19 and others emerging virus-related diseases.

### Future Directions and Concluding Remarks

Multi-OMICs and pharmacogenomics investigations, in line with the concept of precision and tailored medicine, are mandatory in complex disease characterized by a wide range of clinical manifestations **(** Zamboni and Gemmati, 2007; Parmeggiani et al., 2008; Husi et al., 2013; Tisato et al., 2018). A multi-layer approach (COVIDOMICS), also based on integrated novel technologies, can shed light on these crucial points, increasing our knowledge useful against the emergence and spread of future novel viral diseases. This will be strategic for the early detection of at risk populations, to establish adequate preventive measures and targeted therapeutic approaches vaccination program included (Gemmati and Tisato, 2022). Although vaccination represents the best answer against SARS-CoV-2 pandemic, it is evident that the appearance of new variants will represent a permanent challenge for the protection induced by the available vaccines or drugs. In this context, it is a priority to establish as many therapeutic options as possible, to be able to control disease symptoms when vaccination does not provide sufficient protection or when it is not possible. Accordingly, direct and indirect mechanisms of interaction between Sars-CoV-2 S-protein(s) and host-cellular targets may change due to the appearance of new “variants of concern”, potentially leading to different biological outcomes. In this line, dedicated preclinical studies are mandatory to identify how each specific variant might affects these pathways and the degree of mutual unbalancing (Battagello et al., 2020). Restoring the correct balance between p53 and NF-kB represents a promising therapeutic option, particularly in the view of the well-accepted central role of pathways mutual cross talk in the inflammatory settings. To this end, the repositioning of existing drugs (as suggested for Idasanutlin) as well as the development of new active compounds may be of great relevance in the view of synergic treatments able from one side to improve the immune status of individuals and on the other side to target specific pathways involved in disease development and progression.

Finally, in light of the central role of host genetics in determining the individual susceptibility to infection and the risk of worst prognosis, a designed pharmacogenomic approach towards genetically selected pathways aimed at rebalancing the local tissue homeostasis may help a more appropriate treatment selection in line with a personalized medicine.
